# Circadian Pharmacological Effects of Paeoniflorin on Mice With Urticaria-like Lesions

**DOI:** 10.3389/fphar.2021.639580

**Published:** 2022-02-09

**Authors:** Li Peng, Lijuan Wen, Jie Zhang, Xiaotong Zhang, Qin Wei, Jing Guo, Jinhao Zeng

**Affiliations:** ^1^ Hospital of Chengdu University of Traditional Chinese Medicine, Chengdu, China; ^2^ Chengdu University of Traditional Chinese Medicine, Chengdu, China; ^3^ Clinical Skills Center, Hospital of Chengdu University of Traditional Chinese Medicine, Chengdu, China; ^4^ Dermatological Department, Hospital of Chengdu University of Traditional Chinese Medicine, Chengdu, China; ^5^ Geriatric Department, Hospital of Chengdu University of Traditional Chinese Medicine, Chengdu, China

**Keywords:** urticaria-like lesion, paeoniflorin, allergic response, inflammatory cell infiltration, inflammatory cytokine, chronotherapeutic

## Abstract

Paeoniflorin (PF) is a monoterpene glucoside with various biological properties, and it suppresses allergic and inflammatory responses in a rat model of urticaria-like lesions (UL). In the present study, we treated OVA-induced mice presenting UL with PF at four circadian time points (ZT22, ZT04, ZT10, and ZT16) to determine the optimal administration time of PF. The pharmacological effects of PF were assessed by analyzing the scratching behavior; histopathological features; allergic responses such as immunoglobulin E (IgE), leukotriene B4 (LTB4), and histamine (HIS) release; inflammatory cell infiltration [mast cell tryptase (MCT) and eosinophil protein X (EPX)]; and mRNA levels of inflammatory cytokines such as interleukin (IL)-12, IL-6, interferon-γ (IFN-γ), and IL-4. It was demonstrated that PF significantly alleviated scratching behavior and histopathological features, and ZT10 dosing was the most effective time point in remission of the condition among the four circadian time points. Moreover, PF decreased the serum levels of IgE, LTB4, and HIS, and PF administration at ZT10 produced relatively superior effectiveness. PF treatment, especially dosing at ZT10, significantly reduced the number of mast cells and granules and diminished the infiltration of MCT and EPX in the skin tissues of mice with UL. Furthermore, the oral administration of PF effectively decreased the inflammatory cytokine levels of IL-12 mRNA. In conclusion, different administration times of PF affected its efficacy in mice with UL. ZT10 administration demonstrated relatively superior effectiveness, and it might be the optimal administration time for the treatment of urticaria.

## Introduction

Urticaria is characterized by the rapid appearance of pale to bright wheals, erythema, and pruritus on the skin (Schaefer, 2017). Sometimes, the condition is life-threatening and has detrimental effects on the quality of life of patients (Maurer et al., 2011) with angioedema and intense pruritus. Immunoglobulin E (IgE) mediates the accumulation and degranulation of mast cells play a central role in the pathogenesis of urticaria, which results in the release of histamine (HIS) and other inflammatory mediators (Church et al., 2018). Furthermore, urticarial lesions are characterized by lymphocytic infiltration. Antihistamines or monoclonal antibodies are usually prescribed to treat urticaria. However, occasionally, little response, recurrence of symptoms, and side effects such as headache, drowsiness have been observed in some patients (Maurer et al., 2013; Bernstein et al., 2014; Cordeiro Moreira et al., 2016). Moreover, urticaria exacerbates nocturnally, displaying changing patterns in symptom attacks during the day and night (Maurer et al., 2009). Therefore, a safe, effective, selective, and optimal timing of therapy for urticaria has gained attention.

The physiological events and disorders of all creatures on the earth are interlinked to circadian rhythms, such as the sleep-wake cycle, body temperature, immune responsiveness, and anaphylactic reactions (Nakao et al., 2015). Although specific cellular and molecular mechanisms remain largely undefined, the circadian clock was regarded as a potent regulator of IgE/mast cell-mediated allergic reaction (Nakao et al., 2015). Nocturnal symptoms are common in atopic dermatitis and asthma, being related to the diurnal rhythm of inflammatory cell populations (Mackay et al., 1994; Lissoni et al., 1998; Raherison et al., 2006; Fishbein et al., 2015). Moreover, urticaria-like symptoms such as itching, wheals (hives), angioedema, or both, undergo circadian variations, and they exacerbate more frequently at night (Maurer et al., 2009). It has been long recognized that pharmacological effects could be enhanced by time-varying administering drugs of the disease displaying diurnal rhythmicity in severity (Koyanagi et al., 2003). We posit that time-varying drugs may function the treatment efficacy in urticaria.

Paeoniflorin (PF), extracted from the roots of *Paeonia lactiflora* Pall. (Shi et al., 2014; Wang et al., 2015), is a monoterpene glucoside with many biological properties. PF is one of the principal bioactive components of total glucosides, and it has been prescribed for redness, fever, and pain in rheumatoid arthritis patients in China for many years (Wang et al., 2015). Crucially, PF has been found to exhibit the therapeutic effects of various allergic diseases, such as asthma and contact dermatitis in mice. It was demonstrated that PF ameliorated asthma *via* the signaling pathway of phosphoinositide 3 kinase and serine/threonine kinase 1, to regulate the abnormal proliferation and migration of airway smooth muscle cells (Zhou et al., 2018). And PF acts as an immune-modulator to regulate the imbalanced secretion of inflammatory cytokines, such as interleukin (IL)-2, IL-4, IL-10, and IL-17, to treat allergic contact dermatitis in mice (Wang et al., 2015). Additionally, PF inhibits the maturation of dendritic cells and promotes their tolerogenic effects by inhibiting IL-12 production and enhancing the expression of anti-inflammatory cytokines such as IL-10 and transforming growth factor-β (Shi et al., 2014).

In this study, we assessed the ability of PF as a chronotherapeutic to influence the ovalbumin (OVA) induced urticaria-like lesions (UL) in animal models. We explored whether and how the PF exerted effects on histopathological features either in the morning (ZT02) or evening (ZT14) to establish a UL mouse model then dosed at four circadian time points. We also demonstrated whether and how the PF played the medicinal effect on allergic responses among time-varying administering drugs. We identified the role of PF played in the regulation of the Th1 and Th2 expression varied with different dosing time points. Our work may provide experimental evidence for optimal administration time of PF in UL treatment.

## Materials and Methods

### Reagents and Chemicals

Aluminum hydroxide was dissolved in 0.9% NaCl solution to obtain a final concentration of 10 g/L. Furthermore, OVA (5 mg per mouse) (Merck KGaA, Darmstadt, Germany) was added to the mixture to obtain the resulting solution, which will be injected into animals. PF, with a purity quotient of ≥98%, was obtained from the Beijing Solarbio Science and Technology Co., Ltd. (Beijing, China). To obtain the stock solution, PF was dissolved in 50 ml of sterilized 0.9% NaCl at a concentration of 2000 mg/ml (Shi et al., 2015). The working solutions were diluted with saline.

### Animals

One hundred and forty-four BALB/c mice (male) were purchased from Chengdu Dashuo Experimental Animal Co., Ltd. (Chengdu, China), and were housed in the Animal Experimental Center, Chengdu University of Traditional Chinese Medicine (Chengdu, China). All mice were maintained at a temperature of 22 ± 2°C and relative humidity of 55 ± 10% with a 12-h light/dark cycle [lights on at 7:00 am (zeitgeber time (ZT) 0) and lights off at 7:00 pm (ZT12)], and *ad libitum* access to water and food was provided. All mice were 8–9 weeks old, and their body weights were 16–20 g. All procedures were approved by the Institutional Animal Care and Use Committee of Chengdu University of Traditional Chinese Medicine (approval no. 2019-11).

### Induction and Treatment of Animal Models for Urticaria

**FIGURE 1:**
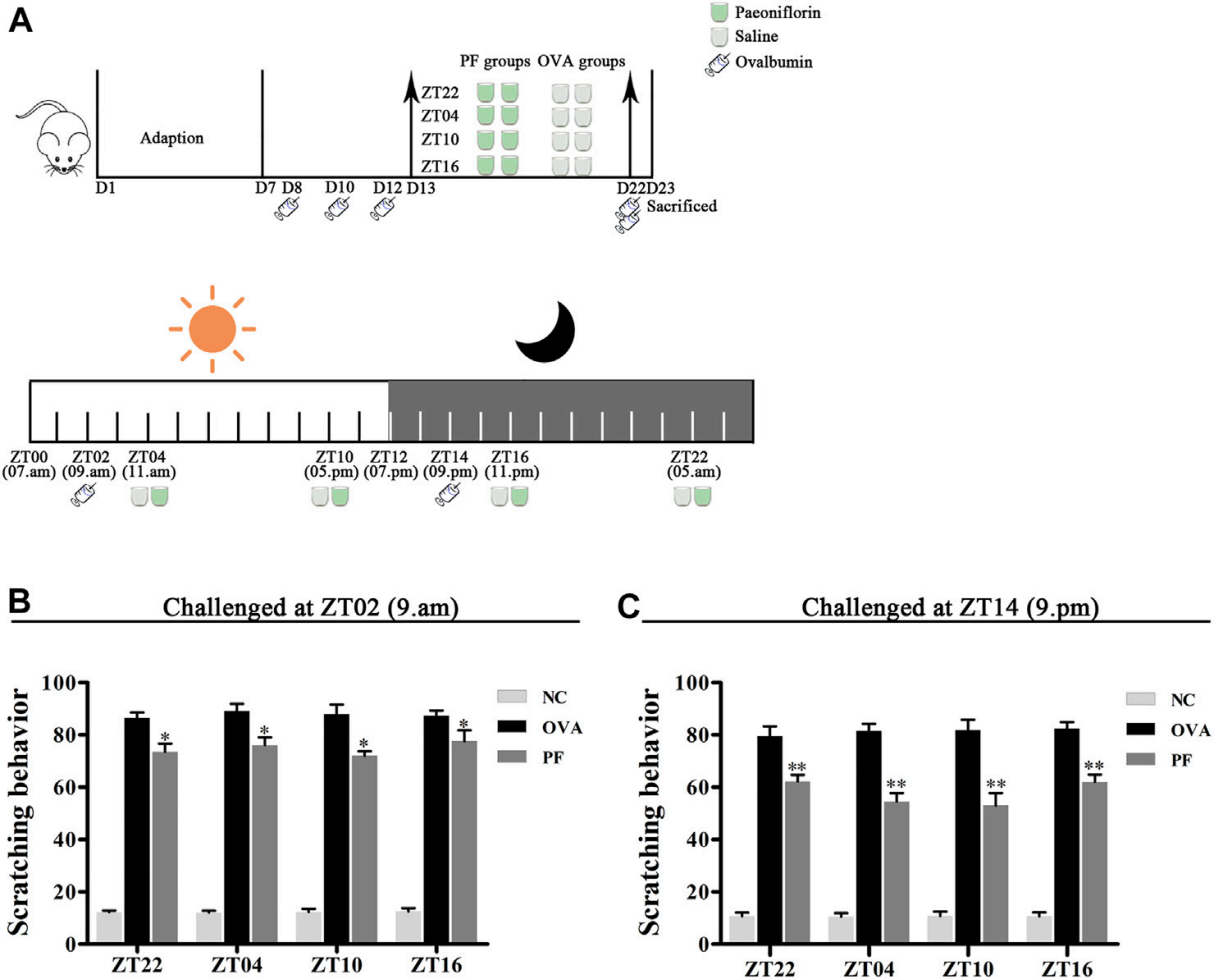
Experimental design **(A)** and Effect of PF treatment on scratching frequency **(B,C)**. 144 male BALB/c mice (urticaria-like animals) were randomly divided into 12 groups as follows modeled at ZT02 or ZT14 (*n* = 6 in each group): normal control (NC) groups treated with saline, including 1) NC ZT22, 2) NC ZT04, 3) NC ZT10, and 4) NC ZT16; model groups injected intraperitoneally (i.p.) with ovalbumin (OVA)/aluminium hydroxide, including 5) OVA + saline ZT22, 6) OVA + saline ZT04, 7) OVA + saline ZT10, and 8) OVA + saline ZT16; and PF groups induced with OVA/aluminium hydroxide and treated with 100 mg/kg per day of paeoniflorin (PF), including 9) OVA + PF ZT22, 10) OVA + PF ZT04, 11) OVA + PF ZT10, and 12) OVA + PF ZT16. To induce urticaria-like lesions (UL), all mice except those in NC groups were sensitized i.p. with 1 ml of aluminium hydroxide solution containing 1 mg of OVA at ZT02 or ZT14 on days 8, 10, and 12, and they were challenged i. p. with 2 mg OVA in aluminium hydroxide at ZT02 or ZT14 on day 22. All animals were orally administered saline or PF solution from day 13 to day 22 for 10 days once a day at ZT22, ZT04, ZT10, and ZT16. As shown in **(A)**, on day 23, the dorsal region of all animals were shaved. After ZT14, 500 µL of the blood sample was collected through the cardiac puncture, and skin tissues were obtained under anesthesia with sodium pentobarbital (60 mg/kg i.p.). Furthermore, animals were humanely euthanized *via* cervical dislocation after sampling. The PF treatment significantly inhibited OVA/aluminium hydroxide-induced scratching reaction. Data are displayed as mean ± SEM (*n* = 6). ^*^ *p* < 0.05, ^**^ *p* < 0.01 versus OVA mice at the same time points.

After 7 days of adaptation, one hundred and forty-four male BALB/c mice were randomly divided into the following groups (*n* = 6 in each group): normal control (NC) groups treated with saline, including 1) NC ZT22, 2) NC ZT04, 3) NC ZT10, and 4) NC ZT16; model groups injected intraperitoneally (i.p.) with OVA/aluminium hydroxide, including 5) OVA + saline ZT22, 6) OVA + saline ZT04, 7) OVA + saline ZT10, and 8) OVA + saline ZT16; and PF groups induced with OVA/aluminium hydroxide and treated with 100 mg/kg of PF per day (Shi et al., 2015), including 9) OVA + PF ZT22, (10) OVA + PF ZT04, 11) OVA + PF ZT10, and 12) OVA + PF ZT16. To induce UL, all mice except the controls were sensitized at ZT02 or ZT14 on days 8, 10, and 12 using an i.p. injection of 1 ml aluminium hydroxide solution containing 1 mg of OVA, and were challenged at ZT02 or ZT14 on day 22 with an i.p. injection containing 2 mg of OVA in aluminium hydroxide solution. All animals were orally administered saline or PF solution from day 13 to day 22 for 10 days once a day at ZT22, ZT04, ZT10, and ZT16. On day 23, the dorsal region of all animals was shaven (Figure 1A). After ZT14, 500 µL of blood samples were collected through the cardiac puncture, and skin tissues were obtained by inducing anesthesia using pentobarbital sodium (60 mg/kg i.p., Merck KGaA, Darmstadt, Germany). Furthermore, animals were humanely authorized *via* cervical dislocation after sampling.

### Assessment of Scratching Behavior

After challenging for 10 min on day 22, the frequencies of scratching and foot licking were measured for 20 min. The lifting of paws; rubbing of dorsal skin, hind paws, nose, or ears; and then returning the paws to the floor is defined as scratching (Choi et al., 2018; Purushothaman et al., 2018).

### Histopathological Analysis

For histopathological observation, the dorsal skin lesions were dissected, dipped in 10% paraformaldehyde (Chengdu Cologne Chemical Co., Ltd., Chengdu, China) overnight, hydrated with ethanol, embedded in paraffin wax (Shanghai Hualing Rehabilitation Equipment Factory, Shanghai, China), and cut into thin sections (3 μm). Skin sections were stained with hematoxylin (Wuhan Seville Biotechnology Co., Ltd., Wuhan, China) and eosin (Zhuhai Bezo Biotechnology Co., Ltd., Zhuhai, China) for detection of edema and inflammatory cell infiltration, images were captured randomly by the pathologist using a light microscope (OLYMPUS, Tokyo, Japan), and they were viewed at a magnification of ×100 (×10ocular lens and ×10 objective lens). The degree of edema, telangiectasia, and inflammatory cell infiltration was scored on a subjective scale of 0–3 as follows which from three fields per hematoxylin and eosin-stained sections: 0, no edema; no telangiectasia; no inflammatory cell infiltration; 1, slight edema; slight telangiectasia; slight inflammatory cell infiltration; 2, moderate edema; moderate telangiectasia; moderate inflammatory cell infiltration; 3, severe edema; severe telangiectasia; severe inflammatory cell infiltration. The histologic score of each animal was the average of the total scores of 3 pathological features from 3 visual fields.

### Determination of Serum IgE, Leukotriene B4 (LTB4), and HIS Levels

Blood samples from mice with UL were centrifuged at 3,000 x g to obtain the serum samples, which were then stored at −80°C until quantitative analysis. The serum levels of IgE, LTB4, and HIS were detected using the respective ELISA kits (ZCI BIO, Shanghai, China), and their optical densities (ODs) were determined using a microplate reader (Shanghai Jizhou chemical industry technology co., ltd., Shanghai, China) at 450 nm according to the manufacturer’s instructions. The concentrations of IgE, LTB4, and HIS were determined based on their standard curves.

### Mast Cell Detection

To evaluate the number and degranulation of mast cells, the skin tissues were fixed with 10% paraformaldehyde (Chengdu Cologne Chemical Co., Ltd., Chengdu, China), dehydrated with ethanol, embedded in paraffin, sectioned, stained with toluidine blue (Shanghai Ruji Biological Technology Development Co., Ltd., Shanghai, China) solution at 37°C for 10 min, and then sealed with a resin. Stained mast cells were enumerated using a microscope (OLYMPUS, Tokyo, Japan) by randomly observing three fields per section at a magnification of ×100 and 400×.

### Detection of Mast Cell Tryptase (MCT) and Eosinophil Protein X (EPX)

After the skin tissues were dewaxed, they were stored in 0.01 mol/L citrate to retrieve the antigen, microwaved for 20 min to expose the antigen, immersed in 0.03% H_2_O_2_ for 15 min to inactivate endogenous peroxidase, and blocked with goat serum blocking solution for 20 min at room temperature. Subsequently, sections were incubated with primary mouse monoclonal anti-MCT antibody (Abcam, Cambridge, United Kingdom) and rabbit polyclonal anti-EPX antibody (Biorbyt, Cambridge, United Kingdom) at 4°C overnight. After rewarming at 37°C for 1 h, samples were washed with phosphate-buffered saline, incubated with a secondary antibody and biotin-labeled goat anti-rabbit IgG at 37°C for 30 min, incubated with biotin-labeled streptavidin at 37°C for 30 min, developed with a DAB kit (ZSGB Bio, Beijing, China), counterstained with hematoxylin, and sealed with a neutral resin. Microphotographs were obtained using a light microscope. Images of each slide were photographed randomly for three fields using a light microscope at ×200 magnification. Image-Pro Plus 6.0 software (MEDIA CYBERNETICS, Maryland, USA) was used to detect the integrated optical density (IOD) of each field. The mean IOD of the three fields was displayed as the semi-quantitative levels of MCT and EPX.

### Measurement of Th2/ Th1 Expression in the Dorsal Skin

The mRNA levels of IL-12, IL-6, interferon (IFN)-γ, and IL-4 in the dorsal tissues were detected using qRT-PCR. Fresh skin tissues were lysed with TRIzol (Hefei Bomei Biotechnology Co., Ltd., Hefei, China) to extract total RNA. PrimeScript RT Reagent Kit (Baori Medicine Biotechnology Co., Ltd., Beijing, China) was used to perform reverse transcription experiments to obtain cDNA for each sample. The cDNA was diluted 10-fold, and it was used as a template for qRT-PCR, which was performed using the Thermo Scientific PikoReal software real-time fluorescent quantitative PCR instrument (Thermo Fisher, Waltham, MA, USA) according to the manufacturer’s instructions. The following cycle was performed 45 times: predegeneration at 95°C for 30 s, degeneration at 95°C for 5 s, annealing at 55°C for 30 s, and extension at 72°C for 30 s. β-actin (Sangon Biotech (Shanghai) Co., Ltd., Shanghai, China), and GAPDH (Wuhan servicebio technology Co., Ltd., Wuhan, China) were used as the reference and internal control mRNA, and the 2^−ΔΔCT^ method was used to count the relative mRNA expression. The primer sequences were used as follows: β-actin, 5′-GAA​GAT​CAA​GAT​CAT​TGC​TCC-3’ (sense) and 5′-TAC​TCC​TGC​TTG​CTG​ATC​CA-3’ (anti- sense); GAPDH, 5′-CCT​CGT​CCC​GTA​GAC​AAA​ATG-3’ (sense) and 5′- TGA​GGT​CAA​TGA​AGG​GGT​CGT-3’ (anti- sense); 5′-CCT​CGT​CCC​GTA​GAC​AAA​ATG-3’ (sense) and 5′-TGA​GGT​CAA​TGA​AGG​GGT​CGT-3’ (anti- sense); IL-12, 5′-TCC​AGC​ATG​TGT​CAA​TCA​CGC​TAC​CT-3’ (sense) and 5′-AGC​CAG​GCA​ACT​CTC​GTT​CTT​GTG​TA-3’ (anti- sense); 5′-CCA​TCA​ACG​CAG​CAC​TTC​AGA-3’ (sense) and 5′-GCT​CAG​ATA​GCC​CAT​CAC​CCT-3’ (anti- sense); IL-6, 5′-TGG​AGC​CCA​CCA​AGA​ACG​ATA​GTC​AA-3’ (sense) and 5′-TGT​CAC​CAG​CAT​CAG​TCC​CAA​GAA​GG-3’ (anti- sense); 5′-CCC​CAA​TTT​CCA​ATG​CTC​TCC-3’ (sense) and 5′-CGC​ACT​AGG​TTT​GCC​GAG​TA-3’ (anti- sense); IL-4, 5′-AGG​AGC​CAT​ATC​CAC​GGA​TGC​GAC​AA-3’ (sense) and 5′-GCG​AAG​CAC​CTT​GGA​AGC​CCT​ACA​G-3’ (anti- sense); 5′-GAT​AAG​CTG​CAC​CAT​GAA​TGA​GT-3’ (sense) and 5′-CCA​TTT​GCA​TGA​TGC​TCT​TTA​GG-3’ (anti- sense); IFN-γ, 5′-GCC​ATC​AGC​AAC​AAC​ATA​AGC​GTC​AT-3’ (sense) and 5′-CCC​GAA​TCA​GCA​GCG​ACT​CCT​TT-3’ (anti- sense); 5′-CTC​AAG​TGG​CAT​AGA​TGT​GGA​AG-3’ (sense) and 5′-TGA​CCT​CAA​ACT​TGG​CAA​TAC​TC-3’ (anti- sense);

### Statistical Analysis

All statistical analyses were performed using SPSS 25.0 software (IBM, New York, USA), and data were presented as mean ± SEM. One-way analysis of variance was used to compare the mean statistical differences between experimental groups at the same time point and between PF groups at different time points. A *p*-value < 0.05 was considered as statistically significant. A factorial design was applied to compare the effects of time and treatment (time × treatment). A *p*-value < 0.05 was considered as statistically significant. Levene’s test was used to assess homoscedasticity, least significant difference test was used to assess the data variance, and Dunnett’s t-test was used to assess unequal variances.

## Results

### Effect of PF on Scratching Behavior

In the scratching test, after being challenged with OVA/aluminium hydroxide solution, an obvious scratching reaction was observed in the mice in the OVA and PF groups at all dosing time points as compared with those in the NC groups both modeled in the morning (ZT02) and evening (ZT14). Compared to the OVA groups, both PF groups demonstrated a significant decrease in scratching number (*p* < 0.05, modeled at ZT02, and *p* < 0.01, modeled at ZT14). However, the PF dose at four circadian time points showed no significance in comparison with each other among the two challenged time points (Figures 1B,C). The scratching behaviors demonstrated that PF relieved the pruritus of the mice with UL, and ZT10 dosing showed the best results (Figures 1B,C).

### Effect of PF on the Histopathological Features and Histologic Scores of UL

**FIGURE 2:**
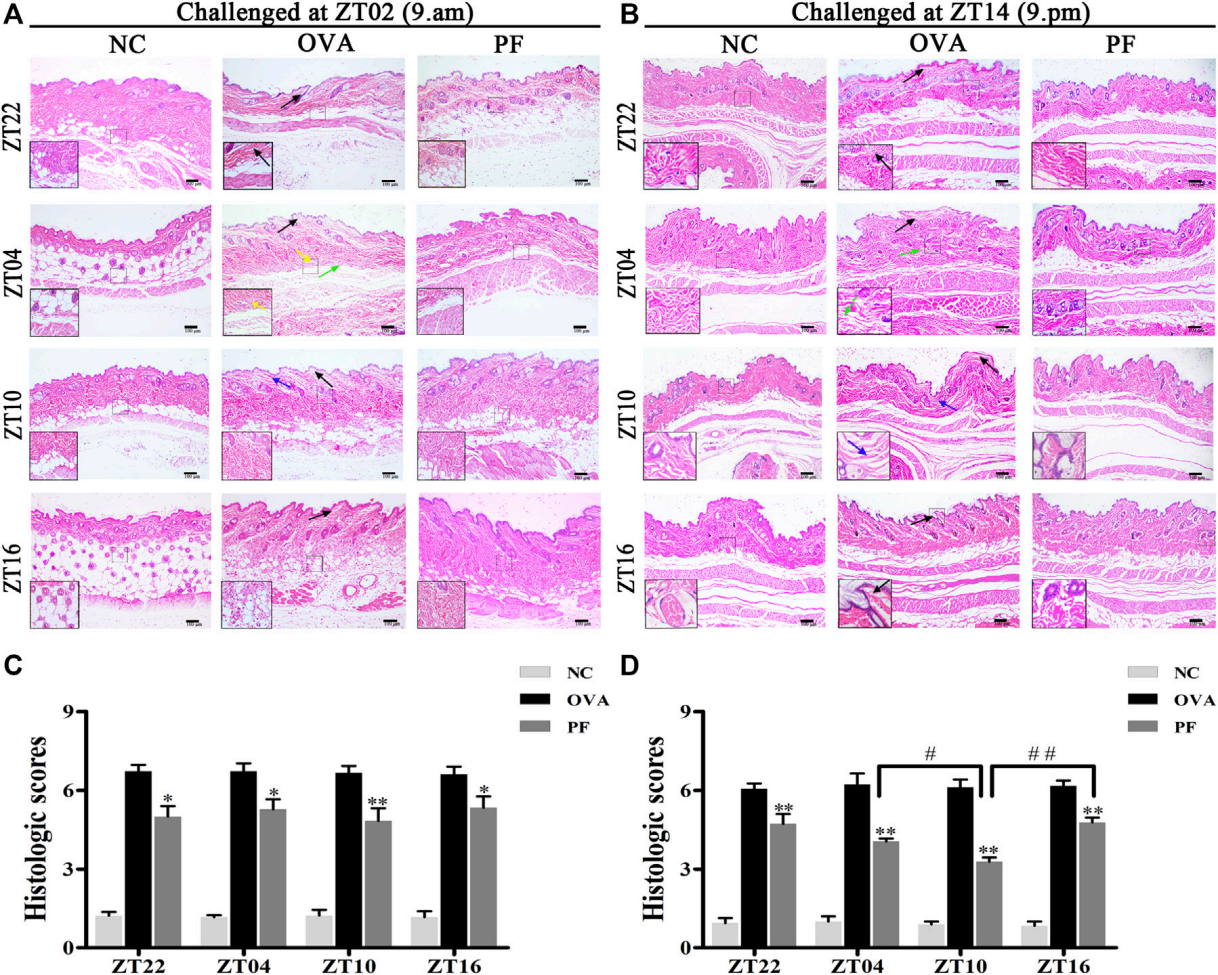
Effect of PF treatment on histopathological features **(A,B)** and histologic scores **(C,D**) in OVA/aluminium hydroxide-induced urticaria-like mice. Representative images of hematoxylin and eosin staining in the dorsal skin. BALB/c mice in the NC group, showing normal architecture. OVA-treated urticaria-like mice showed obvious edema in the upper dermis (black arrows), widening of collagen fiber bundles (blue arrows), with telangiectasia (green arrows), and inflammatory cell infiltration (yellow arrows). PF treatment, especially at ZT10, improved the wheal conditions. The magnification were ×100 and 400×. NC groups have noted less or even no edema, telangiectasia, or inflammatory cell infiltration, OVA groups displayed increased histologic scores, while PF induced significant inhibition of the histologic scores. ^*^ *p <* 0.05, ^**^ *p* < 0.01 versus OVA mice at the same time points. ^#^ *p <* 0.05, ^#*#*^ *p <* 0.01 represents the comparison of PF ZT10 versus the other three PF indicated time points.

Oral administration of PF significantly inhibited the UL in mice induced by OVA/aluminium hydroxide at ZT02 (resting phase) and ZT14 (active phase). Following hematoxylin and eosin staining, compared to the NC groups, obvious edema, mainly in the upper dermis, and widening of collagen fiber bundles with light pink staining was observed in many OVA groups. At the same time, telangiectasia and inflammatory cell infiltration in skin tissues were observed using light microscopy. As expected, PF showed pharmacological effects on the UL in mice at different dosing time points. The dose at ZT10 improved the histopathological conditions as compared with that at other circadian time points regardless of the modeling time in the morning (ZT02) or evening (ZT14) (Figures 2A,B).

The histological findings demonstrated that the NC groups have noted less or even no edema, telangiectasia, or inflammatory cell infiltration while the OVA groups displayed apparent edema, telangiectasia, and inflammatory cell infiltration at all dosing time points, both performed OVA at ZT02 and ZT14. Whereas PF induced significant inhibition of the histologic scores [PF dosing at ZT22 (*p* < 0.05, modeled at ZT02 and *p* < 0.01, modeled at ZT14), PF dosing at ZT04 (*p* < 0.05, modeled at ZT02 and *p* < 0.01, modeled at ZT14), PF dosing at ZT10 (*p* < 0.01, modeled at ZT02 and ZT14), PF dosing at ZT16 (*p* < 0.05, modeled at ZT02 and *p* < 0.01, modeled at ZT14)], compared with the OVA groups among all the dosing time points. Furthermore, when sensitized at ZT14, histologic scores were significantly inhibited by PF dosing at ZT10 (*p* < 0.05 *vs*. ZT04 and *p* < 0.01 *vs*. ZT16, *p* = 0.052 *vs*. ZT22). PF dosing at four indicated time points did not differ significantly among sensitized at ZT02, but ZT10 showed slightly better efficacy than others (Figures 2C,D).

### Effect of PF on the Serum Levels of IgE, LTB4, and HIS

**FIGURE 3:**
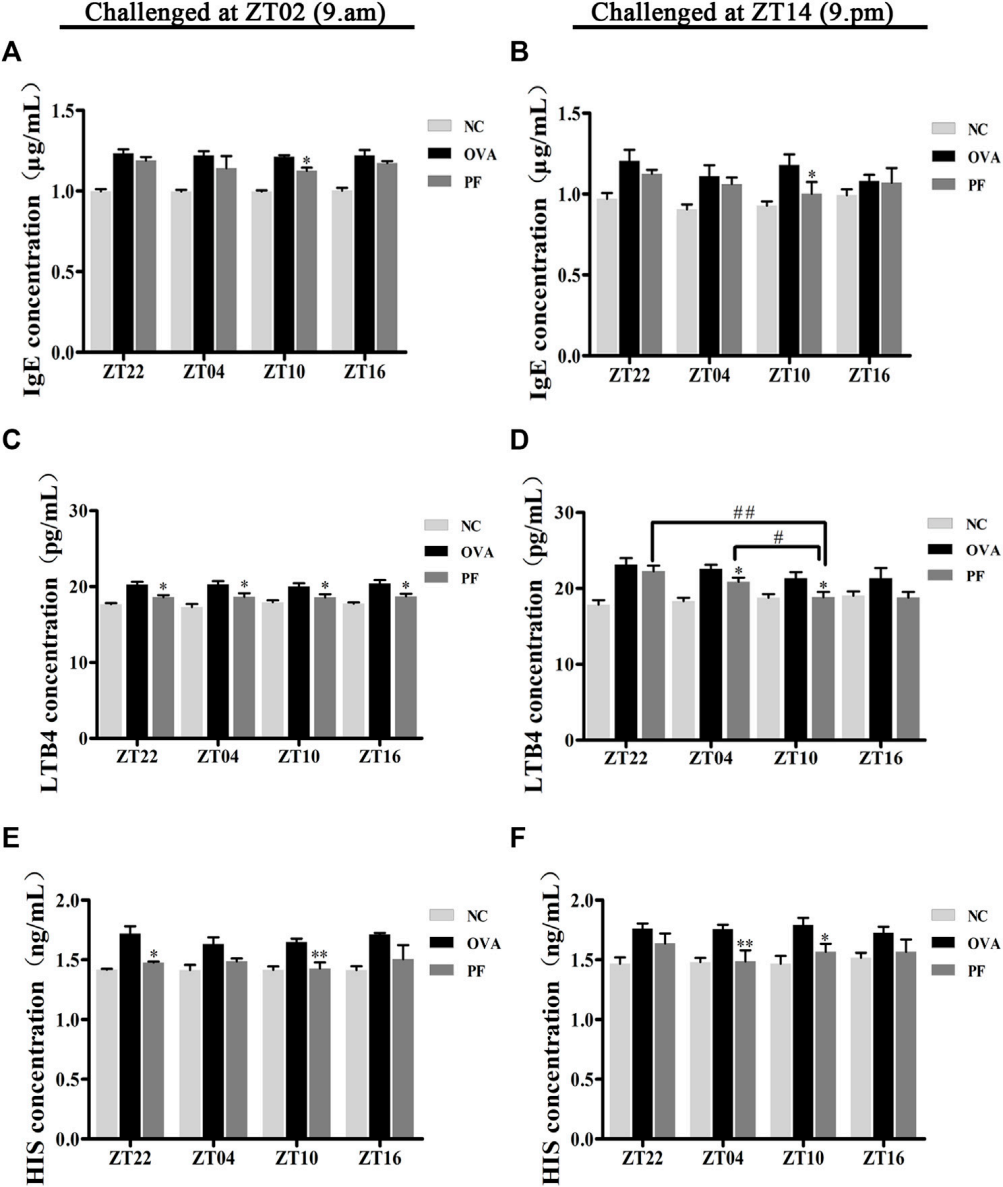
Effect of PF treatment on the secretion of allergic cytokines such as **(A,B)** immunoglobulin E (IgE), **(C,D)** leukotriene B4 (LTB4), and **(E,F)** histamine (HIS) from the serum samples of OVA/aluminium hydroxide-challenged mice. PF downregulated the expression of allergic cytokines. Data are expressed as mean ± SEM (*n* = 6). ^*^ *p <* 0.05, ^**^ *p <* 0.01 versus OVA mice at the same time points. ^#^ *p <* 0.05, ^#*#*^ *p <* 0.01 represents the comparison of PF ZT10 versus the other three PF groups.

Since IgE, HIS, and LTB4 play major roles in the etiology of urticaria, their expression varies greatly with the time of the day. Thus, the study of the effect of PF administration at different time points on anti-allergic response markers is of great interest. The levels of IgE, LTB4, and HIS in the serum of urticaria-like animal models were determined using ELISA. Dosing at ZT22 (*p* < 0.01), modeled at ZT02 and ZT14), ZT04 (*p* < 0.01, modeled at ZT02 and *p* < 0.05, at ZT14), and ZT10 (*p* < 0.01, modeled at ZT02 and ZT14), and ZT16 (*p* < 0.01, modeled at ZT02) increased the IgE levels in OVA groups as compared to those in the NC groups, respectively (Figures 3A,B). A decrease in IgE excretion was observed in PF-treated mice as compared to that of the OVA group at ZT10 (*p* < 0.05, modeled at ZT02 and ZT14) (Figures 3A,B). However, the levels of IgE at the other three circadian time points did not decrease in PF groups statistically significant, compared with the OVA groups (Figures 3A,B). The levels of LTB4 at the four circadian time points were much higher in the OVA groups [ZT22 (*p* < 0.01, modeled at ZT02 and ZT14), ZT04 (*p* < 0.01, modeled at ZT02 and ZT14), ZT10 (*p* < 0.01, modeled at ZT02 and *p* < 0.05, modeled at ZT14), and ZT16 (*p* = 0.004, modeled at ZT02 and *p* = 0.103, modeled at ZT14)] than those in the NC groups. The levels of the serum LTB4 are reduced in both ZT22 (*p* < 0.05, modeled at ZT02), PF ZT04 (*p* < 0.05, modeled at ZT02 and ZT14), and ZT10 (*p* < 0.05, modeled at ZT02 and ZT14), and ZT16 (*p* < 0.05, at ZT02) as compared to the OVA treated mice. However, treatment with PF at ZT22 and ZT16 showed no significant difference in the levels of LTB4 between the OVA groups modeled at ZT14. Furthermore, the PF dosing at ZT10 led to a lower serum level of LTB4 compared to the doses at ZT22 (*p* < 0.01) and ZT04 (*p* < 0.05) modeled in the evening (Figures 3C,D). Compared with the NC groups, the OVA groups showed an increase in the expression of HIS [ZT22 (*p* < 0.05), ZT04 (*p* < 0.05), ZT10 (*p*<0.01), ZT16 (*p*<0.01) modeled at ZT02, ZT22 (*p* < 0.01), ZT04 (*p* < 0.01), ZT10 (*p* < 0.01) modeled at ZT14]. PF ZT22 (*p* < 0.05, modeled at ZT02), ZT04 (*p* < 0.01, modeled at ZT14) and PF ZT10 (*p* < 0.01, modeled at ZT02, and *p* < 0.05, modeled at ZT14) dosing significantly repressed the expression of HIS in the PF groups compared to that in the OVA groups. However, PF-treated at different circadian time points showed no significance in comparison with each other (Figures 3E,F). These results indicate that PF treatment alleviates the allergic conditions of UL mice (Figure 3).

### Effect of PF on Mast Cell Infiltration and Degranulation

**FIGURE 4:**
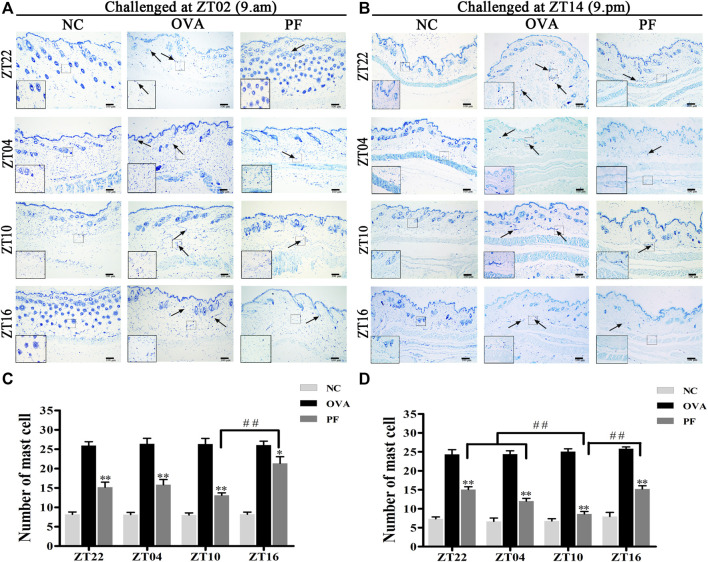
Representative low-magnification light photomicrographs **(A,B)**, and numbers **(C,D)** displaying toluidine blue staining of mast cell degranulation (black arrows). The mast cell degranulation increased with OVA/aluminium hydroxide administration. Oral administration of PF resulted in a significant reduction in mast cell degranulation. Data are expressed as mean ± SEM (*n* = 6). ^*^ *p <* 0.05, ^**^ *p <* 0.01 versus OVA mice at the same time points. ^##^ *p <* 0.01 represents the comparison of PF ZT10 versus the other three PF groups. The magnification were ×100 and 400×.

**FIGURE 5:**
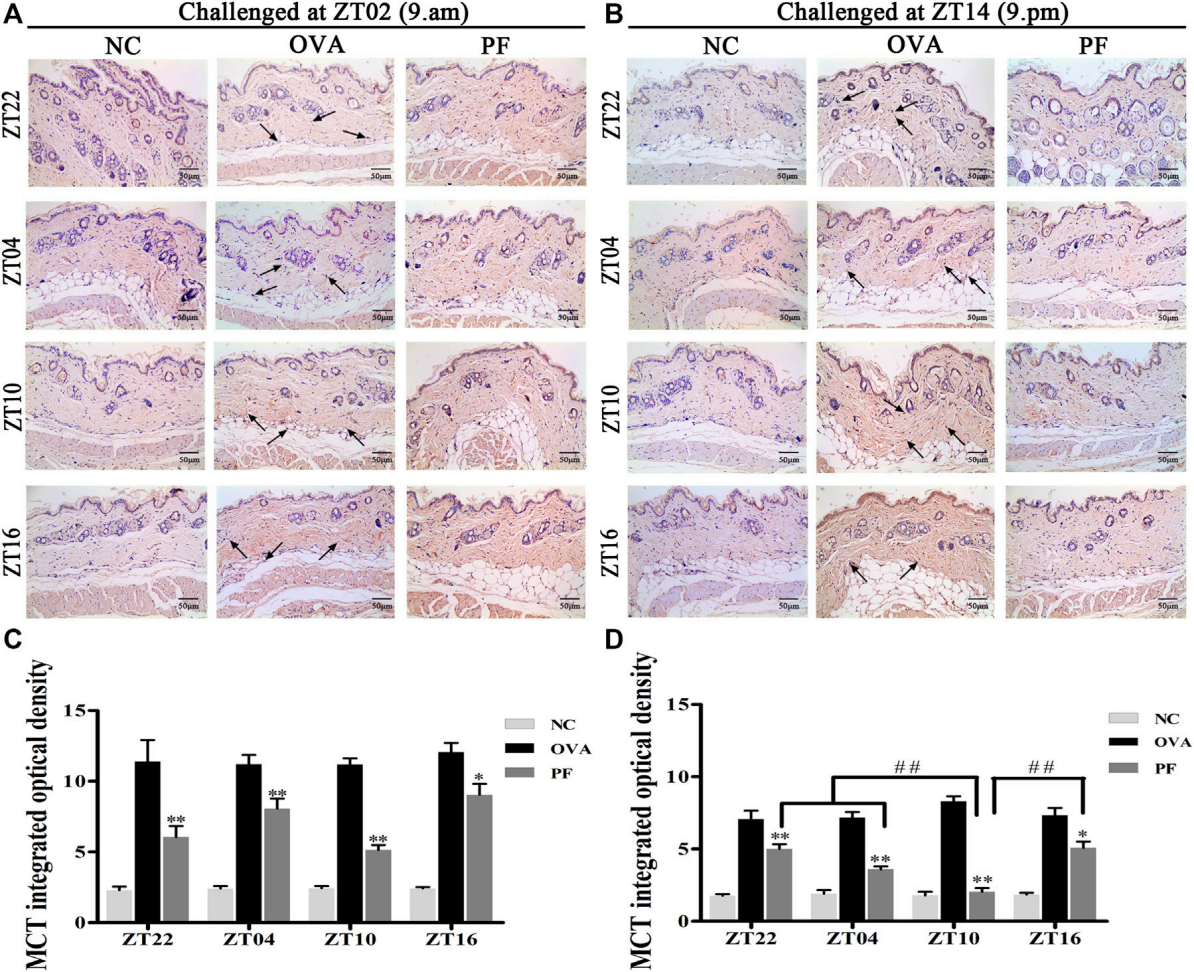
Representative low-magnification light photomicrographs **(A,B)** and integrated optical density **(C,D)** displaying immunohistochemistry of mast cell tryptase (MCT) (black arrows). The PF treatment at ZT10 did possess the best effect on the release of MCT than the other three PF circadian time points. Data are expressed as mean ± SEM (*n* = 6). ^*^ *p <* 0.05, ^**^ *p <* 0.01 versus OVA mice at the same time points. ^##^ *p <* 0.01 represents the comparison of PF ZT10 versus the other three PF groups. The magnification was ×200.

Urticaria is a mast cell-driven disease. MCT is the most abundant serine protease in mast cells, with mature and immature forms. In urticaria, activated mast cells secrete mature tryptase. One of the biological functions of MCT is correlated with pro-inflammatory effects, involving tissue edema and remodeling, chemokine secretion, and neutrophil recruitment. To determine the infiltration and degranulation of mast cells, we performed toluidine blue staining (Figures 4A,B) to analyze the numbers and degranulation of mast cells and immunohistochemistry to determine the IOD of MCT (Figures 5A,B). The OVA challenged in the morning and evening [ZT22 (*p* < 0.01), ZT04 (*p* < 0.01), ZT10 (*p* < 0.01), and ZT16 (*p* < 0.01)] resulted in a marked and significant increase of mast cell granules compared with the observations in the NC groups. Treatment of mice with PF resulted in a significant reduction in the number of mast cells and granules at ZT22 (*p* < 0.01 *vs*. OVA), ZT04 (*p* < 0.01 *vs*. OVA), ZT10 (*p* < 0.01 *vs*. OVA), with modeling at ZT02 and ZT14, and ZT16 (*p* < 0.05 *vs*. OVA, modeled at ZT02, and *p* < 0.01 *vs*. OVA, modeled at ZT14). At ZT10, PF induced a significant reduction [(*p* < 0.01 *vs*. ZT16) modeled at ZT02, (*p* < 0.01 *vs*. ZT22), (*p* < 0.01 *vs*. ZT04), and (*p* < 0.01 *vs*. ZT16) modeled at ZT14] in mast cell granules in comparison with other three circadian time points (Figures 4C,D). The results of MCT were consistent with these results in the OVA groups [ZT22 (*p* < 0.01 *vs*. NC), ZT04 (*p* < 0.01 *vs*. NC), ZT10 (*p* < 0.01 *vs*. NC), and ZT16 (*p* < 0.01 *vs*. NC)] with modeling in the morning and evening. Compared to the OVA groups, PF [ZT22 (*p* < 0.01), ZT04 (*p* < 0.01), ZT10 (*p* < 0.01), and ZT16 (*p* < 0.05) modeled at ZT02 and ZT14] significantly decreased the IOD of MCT. The PF dosing at ZT10 [ZT22 (*p* < 0.01), ZT04 (*p* < 0.01), and ZT16 (*p* < 0.01), modeled at ZT14] displayed the best treatment efficacy to decrease the IOD of MCT (Figures 5C,D).

### Effect of PF on EPX in Skin Tissues

**FIGURE 6:**
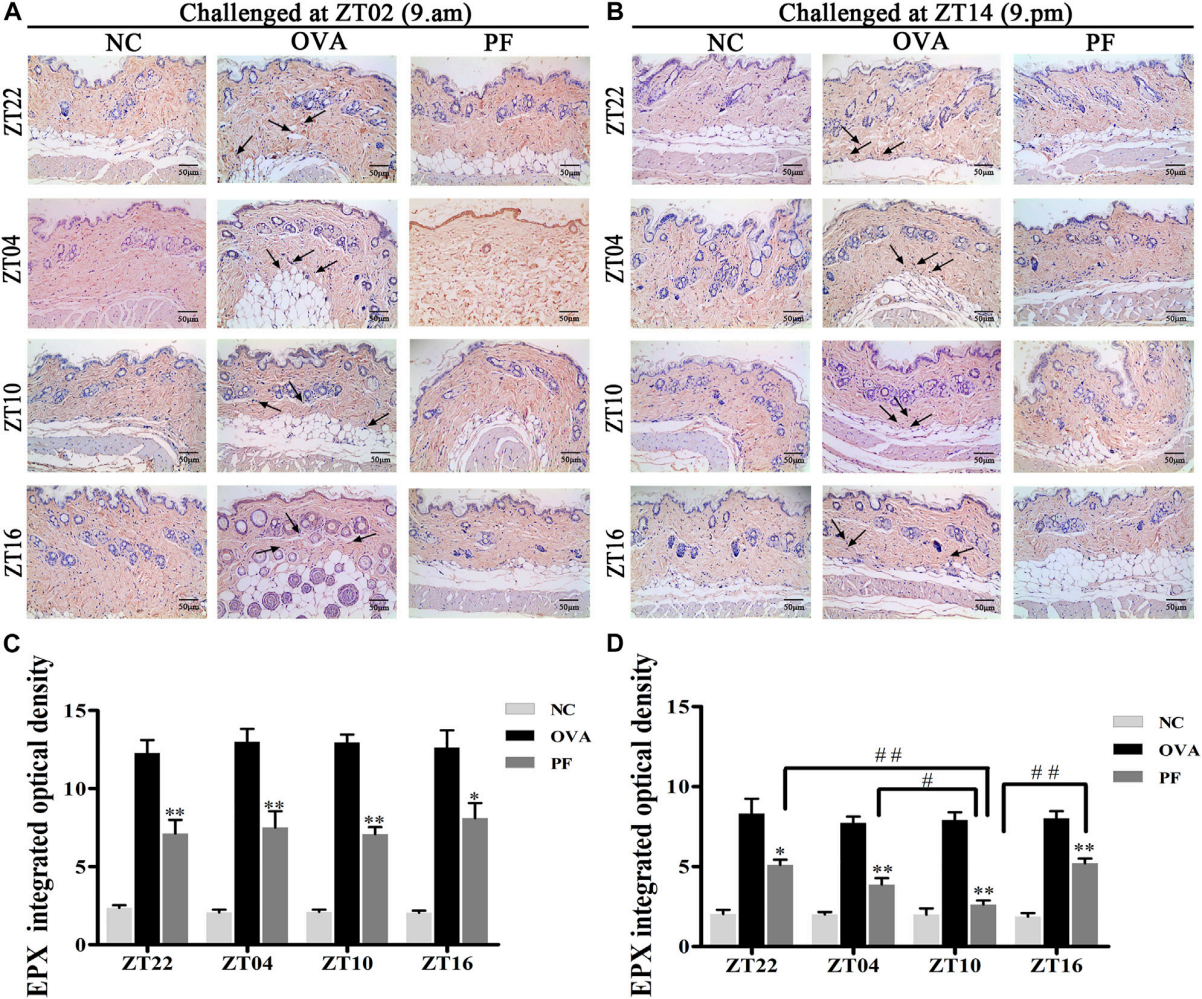
Representative low-magnification light photomicrographs **(A,B)** and integrated optical density **(C,D)** displaying immunohistochemistry of eosinophil protein X (EPX) (black arrows). The PF treatment at ZT10 had a greater effect on EPX production. Data are expressed as mean ± SEM (n = 6). *p < 0.05, **p < 0.01 versus OVA mice at the same time points. #*p* < 0.05, ##*p* < 0.01 represents the comparison of PF ZT10 versus the other three PF groups. The magnification was ×200.

To assess the effect of dosing time on EPX, mice with UL were administered PF using an oral gavage at four circadian time points (ZT16, ZT22, ZT04, and ZT10). EPX as an eosinophilic marker, and released by activated eosinophils, which function in immune responses (Weller and Spencer, 2017; Kim et al., 2020). The IOD of EPX in the OVA groups both for the two challenged time points [ZT22 (*p* < 0.01), ZT04 (*p* < 0.01), ZT10 (*p* < 0.01), and ZT16 (*p* < 0.01)] was significantly elevated at the four circadian time points compared to that in NC groups. However, the expression levels of EPX reduced in all circadian time groups after treatment with PF [ZT22 (*p* < 0.01), ZT04 (*p* < 0.01), ZT10 (*p* < 0.01), and ZT16 (*p* < 0.05) modeled at ZT02 and ZT22 (*p* < 0.05), ZT04 (*p* < 0.01), ZT10 (*p* < 0.01), and ZT16 (*p* < 0.01), modeled at ZT14]. Furthermore, PF ZT10 [(*p* < 0.01 *vs*. ZT22), (*p* < 0.05 *vs*. ZT04), and (*p* < 0.01 *vs*. ZT16), modeled at ZT14] dosing was more effective in decreasing the IOD levels of EPX (Figures 6A–D).

### Effect of PF on the mRNA Levels of IL-12, IL-6, IFN-γ, and IL-4

**FIGURE 7:**
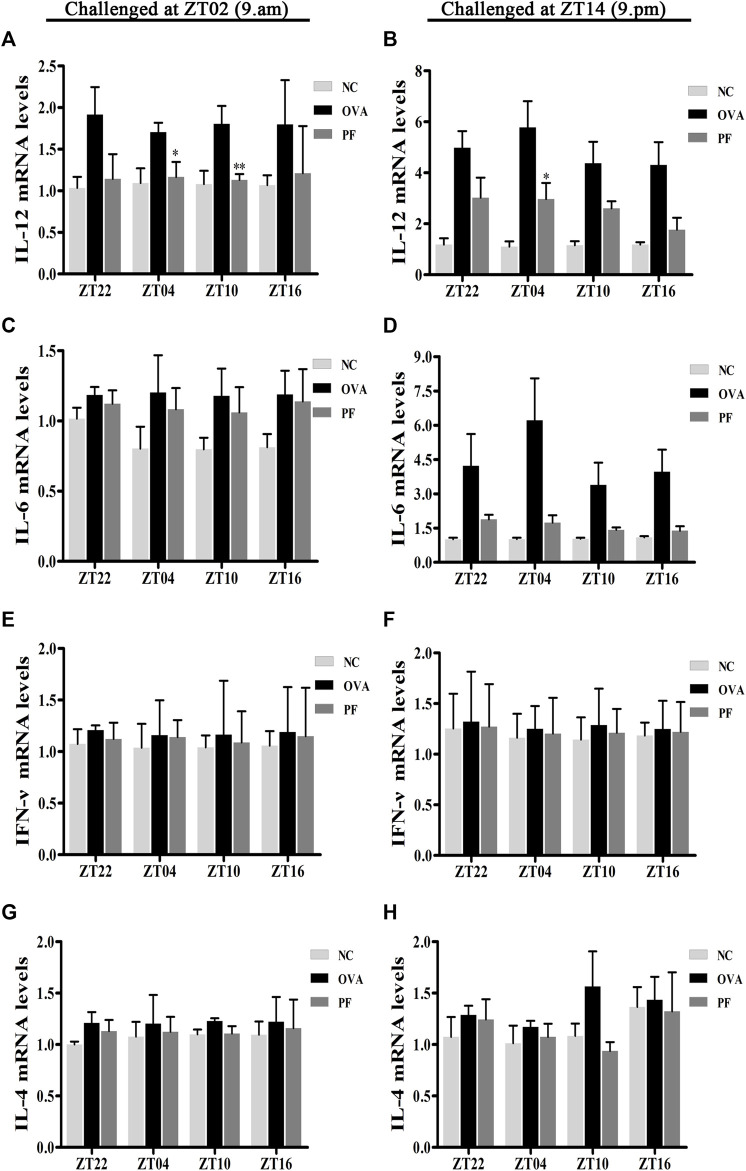
Effect of PF treatment on the release of inflammatory cytokines such as **(A,B)** interleukin (IL)-12, **(C,D)** IL-6, **(E,F)** interferon (IFN)-γ, and **(G,H)** IL-4 from the skin tissues of OVA/aluminium hydroxide-challenged mice. PF reduced the expression of IL-12 mRNA at ZT04 and ZT10. Data are expressed as mean ± SEM (*n* = 6). ^*^ *p* < 0.05, ^**^ *p* < 0.01 versus OVA mice at the same time points.

To elucidate the effects of PF on the inflammatory cytokine release in urticaria, we measured the mRNA levels of IL-12, IL-6, IFN-γ, and IL-4 in skin lesions using qRT-PCR. Dosing at ZT22 (*p* < 0.05), ZT04 (*p* < 0.05), ZT10 (*p* < 0.01) modeled at ZT02, and dosing at ZT22 (*p* < 0.01), ZT04 (*p* < 0.01), and ZT10 (*p* < 0.05), ZT16 (*p* < 0.05) modeled at ZT14 expressed a higher IL-12 mRNA level in the OVA groups with UL as compared to that in the NC groups (Figures 7A,B). However, the expression of IL-12 mRNA at PF ZT04 (*p* < 0.05, modeled at ZT02), ZT10 (*p* < 0.01, modeled at ZT02), and PF ZT04 (*p* < 0.05, modeled at ZT14) decreased as compared to that in the OVA-treated animals (Figures 7A,B). As compared with OVA-treated, no significant differences of PF-treated were observed in the other dosing circadian time points. Additionally, no significant difference was observed in the PF groups between the dosing at ZT16, ZT22, ZT04, and ZT10. For the two indicated model time points, the IL-6 mRNA levels were higher in the OVA-induced mice than that in the NC mice. Moreover, PF treatment at the four dosing circadian time points reduced the levels of IL-6 mRNA. However, the results were not statistically significant (Figures 7C,D). The levels of IFN-γ mRNA elevated in OVA-induced UL mice, and they were reduced after PF administration. However, the data presented no statistical significance (Figures 7E,F). The levels of IL-4 mRNA showed no statistical difference in OVA-induced UL mice when compared to the levels in the NC groups at the four circadian time points. When compared with the OVA groups, PF groups showed no administration time-dependent effects on regulating the expression of IL-4 mRNA (Figures 7G,H).

## Discussion

The prevalence of chronic urticaria ranges from 0.5 to 5% among the general population, leading to a heavy economic burden on the patients. At 19 hospitals in different provinces of China, 60% of urticaria patients experienced their nocturnal attack as reported (Zhong et al., 2014). Nocturnal symptoms attack predominantly around 9 p.m. (ZT14), from another statistical data of 352 patients in China (Huang et al., 2016). The mice were sensitized and challenged at ZT14 to be synchronized with nocturnal symptoms of humans. In our study, the pharmacological effects of PF on urticaria-like symptoms varied with different dosing time points, and PF dosing at ZT10 demonstrated better effects than that at other circadian time points.

Urticaria, a common immune-mediated inflammatory skin disease, causing edema and itching, is associated with IgE-mediated mast cell degranulation and histamine release. IgE is regarded as a potential trigger for the development of urticaria due to its ability to transfer allergen reactivity (Galli and Tsai, 2012; Platts-Mills et al., 2016) and stimulate the degranulation of mast cells. Activated mast cells can secrete pro-inflammatory triggers such as HIS, prostaglandin E- 2, and LTB4 to initiate and amplify hypersensitivity (Galli et al., 2005). MCT, released by mast cells during degranulation, is regarded as a pro-inflammatory mediator in allergic diseases. EPX is a cytotoxic preformed protein, released by the potent pro-inflammatory cells, eosinophil granulocytes. High concentrations of EPX can maintain the secretory activity of mast cells by dissolving mast cells and binding the dissolved matter (Henderson et al., 1980). Injection of OVA and aluminum hydroxide solution stimulated UL in mice at ZT02 or ZT14, leading to an increase in scratching behavior, histopathological changes such as edema, inflammatory cell infiltration, increase in the levels of IgE, HIS, and LTB4, enhance the number of mast cell granules, and concentration of MCT and EPX. These results concur with published data in the literature (Goto et al., 1997; Gu et al., 2004; Hayashi and Fujii, 2008). In addition, abnormalities of the above-mentioned indicators in UL mice were reduced after PF treatment, especially at ZT10.

The circadian rhythm is exhibited by almost all tissues and organs in our body, and disorders in the rhythm induce diseases including urticaria (Smolensky et al., 2015). Metabolic, endocrine, and behavioral functions are governed by circadian rhythms. The number of lymphocytes, T cells, and EPX in human peripheral blood and activation of mast cells exhibit nocturnal peak levels (Pelegrí et al., 2003; Wolthers and Heuck, 2003; Baumann et al., 2013). Pro-inflammatory cytokine levels of IL-12 and IFN-γ are high in the late evening or early morning in human blood (Petrovsky et al., 1998). In addition, blood histamine peaks at the end of the light phase and maintains throughout the dark phase in rats (Friedman and Walker, 1969). These modifications may be responsible for nighttime symptoms, such as itching, wheals.

In our study, oral administration of PF once a day exerted therapeutic effects on mice with UL, although pharmacokinetic study revealed that PF with characteristics of fast absorption rate, short retention time, and low bioavailability (below 5%) (Wang et al., 2008; Wu et al., 2009). *In vivo*, PF is distributed rapidly and widely in various tissues such as the stomach, intestine, spleen, pancreas, and ovary in rats after oral administration of total glycosides of paeony. Besides, the amount of drug in immune organs, such as the spleen and thymus, is greater than in plasma, suggesting that PF may have immune-modulating effects *in vivo* (Fei et al., 2016). Studies also provided the most promising evidence that PF has been widely used as the immune-regulatory agent in immune disorder diseases, including inflammatory bowel disease, psoriasis, asthma, and so on (Gu et al., 2017; Yu et al., 2017; Shou et al., 2018).

Previous studies have reported an imbalance in Th1 and Th2 cells contribute to the progression of UL. IL-4 potentially triggers and sustains mast cells, programs the Th2 cytokine differentiation (Morawetz et al., 1996), and switches B cells to the IgE isotype to induce allergic reactions (Bradding, 1996). IFN-γ, a Th1 effector cytokine, is related to the mitigation of allergic diseases (Teixeira et al., 2005). It is well known that IL-12 promotes Th1 cell polarization, which is associated with the secretion of IFN-γ by T cells (Youssef et al., 2002; Cho et al., 2010). The levels of IL-4, IFN-γ, and IL-6 significantly increase in the dorsal skin of animals with inflammatory diseases such as atopic dermatitis (Choi et al., 2018). In our study, the level of IL-12 mRNA, IL-6 mRNA, IFN-γ mRNA, and IL-4 mRNA were on the rise in UL mice. And PF administration produced a decreasing trend of the cytokines, although our data showed no significance. The persistence of Th1 or Th2 cells predominate is the leading cause of chronic course in some diseases. Th1 cells have been reported to persist in chronic inflammatory diseases, are potent inducers of inflammation due to being repeatedly activated (Niesner et al., 2008; Yamada et al., 2008). And persistent Th2 cells infiltration was associated with the allergic responses of asthma (Wells et al., 2009). Th1 and Th2 are the effector cell subsets of CD4+T cells through the differentiation, which were promoted by cytokines (Mosmann et al., 1986). Furthermore, memory CD4+T cells retain a lifelong ability to mediate protective immunity after vaccination in lymphoid tissues (Amara et al., 2004; Lees and Farber, 2010). To sum up, regulating CD4+T cells is essential for their long-lasting property. Considering the high concentration of PF in the immune organs, and PF-induced regulatory effects on the imbalance in Th1 and Th2 cells, we speculated that PF may exert a continuing medicinal effect through immunoregulatory.

The literature recorded that the intestine could absorb an amount of PF, the unabsorbed fraction is mainly metabolized in intestinal bacteria (Takeda et al., 1997). Bacteroide is one of the intestinal bacteria, playing an important role to convert PF to metabolites (Hattori et al., 1985). It has been reported that gut characteristic metabolite of PF (Benzoic acid) could through the blood-brain barrier to alleviate depressive symptoms in the central nervous system (Yu et al., 2019). Furthermore, gut microbiota and the immune system interact with each other (Kehrmann et al., 2020). *Bacteroides fragilis*-derived Polysaccharide A can differentiate CD4+T-cell into Treg cells, which are a major role in decreasing inflammation (Lahl et al., 2007; Round and Mazmanian, 2010). Another significant aspect is that the majority of the bacterial strains persist for years or even for a lifetime in the host body, which may influence biological functions. In addition, microbiota changed over time, but, more than 70% of bacterial strains remain stable over 1 year for the individual’s microbiota (Faith et al., 2013). The evidence implied that gut microbiota may contribute to the therapeutic role of PF on UL mice persist for some time, which warrants further development.

The activation of mast cells, lymphocytes, T cells, EPX, and cytokines exhibit circadian variations in allergic reactions, showing a high level at night than day. On the other hand, PF is distributed rapidly in the stomach and intestine, the absorption capability of which was high at night compared with day in mice (Hussain and Pan, 2009; Pan and Hussain, 2009). The abundance of bacteroidetes experience diurnal oscillation, peaking at ZT16, troughing around ZT0 in mice, continue to rise during ZT0 to ZT8, slightly fall to ZT12, then rise to ZT16 (Liang et al., 2015). PF dosing at ZT10, therefore, experienced a period of relatively longer detention time to be absorbed then underwent the phase of abundant metabolites producing, which may reinforce the effect. The above information provided some explanation to dosing at ZT10 demonstrated better effects on prophylaxis and treatment than others before symptom onset.

The primary outcome of interest in our experiment was the efficacy of PF on the nocturnal seizures with urticaria, the mice were therefore sensitized and challenged at ZT14 so as to mimic nocturnal symptoms. In order to get more detailed information about antigen challenge and phenotype evaluation at different circadian time points, we performed OVA challenged at ZT02 corresponding to ZT14 so as to imitate morning symptoms of urticaria, and then administrated at ZT22, ZT04, ZT10, and ZT16. The supplementary experiment indicated that the symptoms, histopathological features, mast cell infiltration were severe during the morning, and they were resolved after PF treatment. PF dosing at ZT10 demonstrated better efficacy in comparison to the other three treated indicated time points. Once more, these results provided strong evidence that PF administration at ZT10 produced relatively superior effectiveness to the urticaria symptoms (Figure 1B; Figures 2A,C; Figures 3A,C,E; Figures 4A,C; Figures 5A,C; Figures 6A,C; Figures 7A,C,E,G).

The present study preliminarily revealed the circadian pharmacological effects of PF on mice with UL. However, there were some limitations noted. For instance, detailed mechanisms underlying the circadian pharmacological effects of PF remain to be elucidated. Moreover, since the pathogenesis of urticaria involves a complex multistep process which is affected by many target cells, we wound investigate the mechanism of the optimal administration time of PF specific to a single target cell in subsequent studies. The expression of IL-6 mRNA, IFN-γ mRNA, IL-4 mRNA showed no statistical differences, which may be due to the small sample size or the possibility that the cytokines were not the targets for PF-induced therapeutic effects.

In summary, the symptoms, pathologies factors, and metabolism express diurnal rhythmicity may be the reasons for the difference induced among circadian PF administration timing. In our study, oral administration of PF at four circadian time points (ZT22, ZT04, ZT10, and ZT16) alleviated UL mice, including pruritus, histopathological changes, decreased the levels of IgE, LTB4, and HIS, mast cell infiltration, MCT, EPX, and regulated disorders of Th1 and Th2 cells. It is worth noting that PF administration at ZT10 produced relatively superior effectiveness regardless of whether the UL symptoms attack in the morning or the evening. PF may be used as a potential natural alternative for the prevention and treatment of urticaria before their nocturnal symptoms outbreak.

## Data Availability

The datasets presented in this study can be found in online repositories. The names of the repository/repositories and accession number(s) can be found in the article/Supplementary Material.

## Abbreviations

EPX, eosinophil protein X; HIS, histamine; IgE, Immunoglobulin E; IL, interleukin; IOD, integrated optical density; IFN, interferon; i.p, intraperitoneally; LTB4, leukotriene B4; MCT, mast cell tryptase; NC, normal control; OVA, ovalbumin; PF, paeoniflorin; UL, urticaria-like lesions; ZT, zeitgeber time.
