# Corrigendum: Circadian Pharmacological Effects of Paeoniflorin on Mice With Urticaria-like Lesions

**DOI:** 10.3389/fphar.2022.918401

**Published:** 2022-05-19

**Authors:** Li Peng, Lijuan Wen, Jie Zhang, Xiaotong Zhang, Qin Wei, Jing Guo, Jinhao Zeng

**Affiliations:** ^1^ Hospital of Chengdu University of Traditional Chinese Medicine, Chengdu, China; ^2^ Chengdu University of Traditional Chinese Medicine, Chengdu, China; ^3^ Clinical Skills Center, Hospital of Chengdu University of Traditional Chinese Medicine, Chengdu, China; ^4^ Dermatological Department, Hospital of Chengdu University of Traditional Chinese Medicine, Chengdu, China; ^5^ Geriatric Department, Hospital of Chengdu University of Traditional Chinese Medicine, Chengdu, China

**Keywords:** urticaria-like lesion, paeoniflorin, allergic response, inflammatory cell infiltration, inflammatory cytokine, chronotherapeutic

In the original article, there was a mistake in “Figure 6” as published. Due to the large number of images being processed, one of them was incorrectly processed and uploaded, resulting in its erroneous inclusion within Figure 6. What’s more, according to university and hospital new regulations, multiple funds cannot be written in one manuscript, so we retained the first 3 grants. The corrected Funding statement is “This work was supported by the National Natural Science Foundation of China (grant nos. 82074443 and 81873310), the Sichuan Provincial Central Government Leading Local Science and Technology Development Special Project (grant nos. 2021ZYD0089).” The corrected “Figure 6” appears below. After checking, we have found there was no problem with the statistical analysis.

**FIGURE 6:**
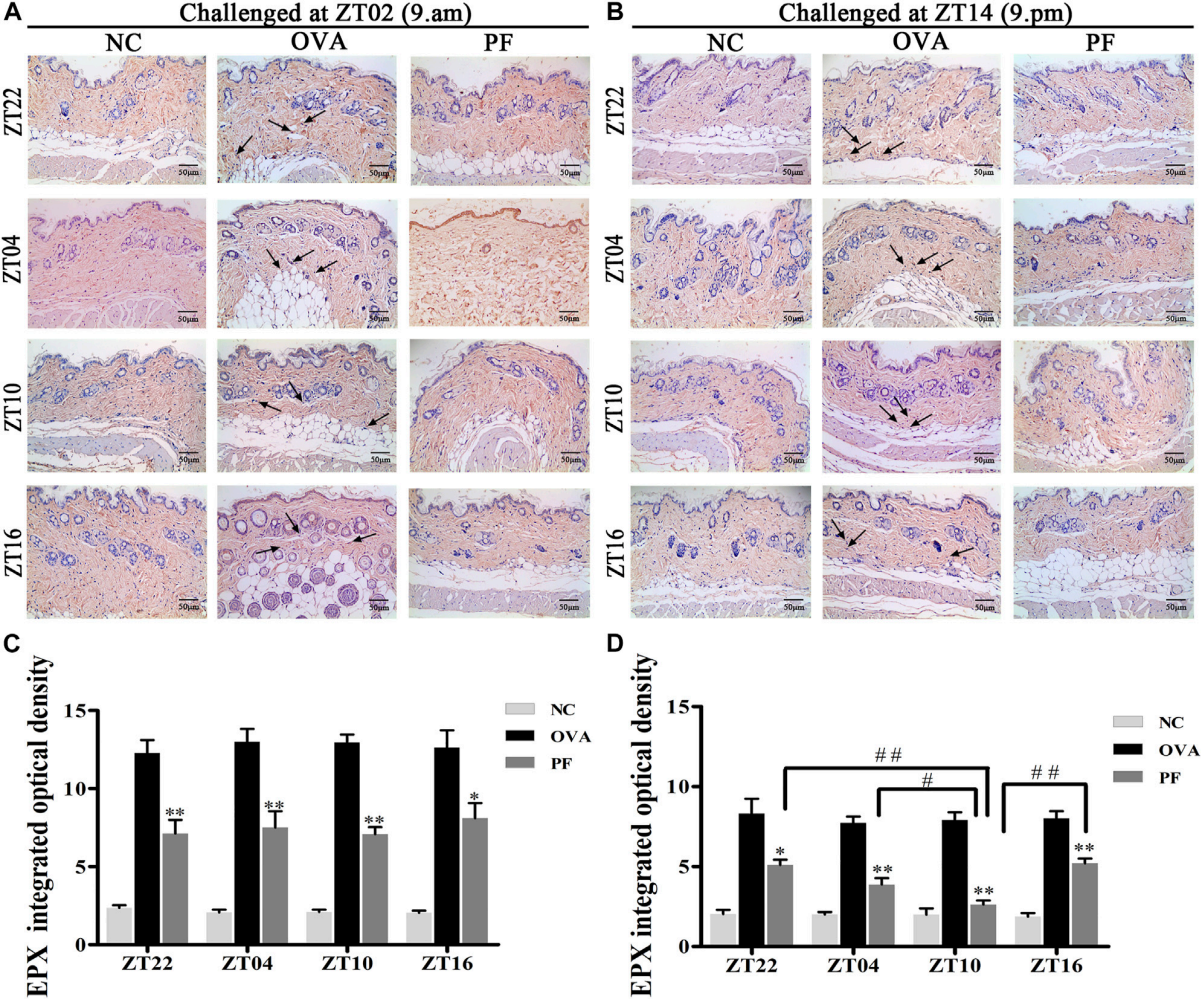
Representative low-magnification light photomicrographs **(A,B)** and integrated optical density **(C,D)** displaying immunohistochemistry of eosinophil protein X (EPX) (black arrows). The PF treatment at ZT10 had a greater effect on EPX production. Data are expressed as mean ± SEM (*n* = 6). *p < 0.05, **p < 0.01 versus OVA mice at the same time points. ^#^ *p* < 0.05, ^##^ *p* < 0.01 represents the comparison of PF ZT10 versus the other three PF groups. The magnification was ×200.

The authors apologize for this error and state that this does not change the scientific conclusions of the article in any way. The original article has been updated.

